# Complete genome sequence of *Selenomonas species* strain TAMA-11512, isolated from blood culture of a septic patient

**DOI:** 10.1128/mra.00070-24

**Published:** 2024-03-11

**Authors:** Kazuhiro Horiba, Sakura Aso, Rentaro Oda, Yoshinori Tateishi, Kuniko Ura, Makoto Kuroda

**Affiliations:** 1 Laboratory of Bacterial Genomics, Pathogen Genomics Center, National Institute of Infectious Diseases, Tokyo, Japan; 2 Department of Clinical Laboratory, Tokyo Metropolitan Tama Medical Center, Tokyo, Japan; 3 Department of Infectious Diseases, Tokyo Metropolitan Tama Medical Center, Tokyo, Japan; Department of Biological Sciences, Wellesley College, Wellesley, Massachusetts, USA

**Keywords:** *Selenomonas*, sepsis, whole-genome sequencing, clinical isolate

## Abstract

We report the complete sequence of *Selenomonas* species strain TAMA-11512, isolated from the blood culture of a septic patient. The phylogeny and average nucleotide identity show that the strain TAMA-11512 is considered a novel bacterial species in *Selenomonas* genus.

## ANNOUNCEMENT

*Selenomonas* species has the properties of anaerobic, Gram-negative, curved, or crescent-shaped rods with flagella originating from the inner curvature of the cell (1). It has been isolated from the human oral cavity and mammal gastrointestinal tract. The involvement of oral anaerobes, including oral selenomonads, as potential pathogens in patients with decreased host defenses was reported (2). Whole blood from the septic patient was cultured using two sets of blood culture aerobic/anaerobic bottles and an automated blood culture system, BACT/ALERT VIRTUO (bioMérieux, Lyon, France) in Tokyo Metropolitan Tama Medical Center in 2023. The anaerobic bottle was positive at 114.9 hours of incubation. From the anaerobic bottle solution, the isolated strain, TAMA-11512, was grown on ABHK agar plates after 48 hours’ incubation anaerobically at 35°C. Bacterial DNA was extracted using Genomic-tip 100 /G columns (Qiagen, Hilden, Germany). Sequencing libraries were prepared using a QIAseq FX DNA library kit (Qiagen) for Illumina sequencing and a Native Barcoding Kit 96 (SQK-NBD112.96; Oxford Nanopore Technologies, Oxford, UK) for Nanopore sequencing, according to the manufacturer’s instructions. Illumina sequencing and Nanopore sequencing were performed using an iSeq 100 system (Illumina, San Diego, CA, USA) with a 2  ×  150 bp paired-end protocol and a GridION platform (Oxford Nanopore Technologies) using R10.4 flow cell (FRO-MIN112), respectively. For Nanopore sequencing, MinKNOW (version 22.12.5) and Guppy (version 6.4.6) (Oxford Nanopore Technologies) were used for base calling and adapter trimming of the raw data. Quality filtering was performed using Fastp (version 0.23.2) (3) for Illumina sequencing data and NanoFilt (version 2.8.0) (4) for Nanopore sequencing data. After quality filtering, the processed Illumina/Nanopore sequencing reads, which mapped to the *Ovis aries* (sheep) National Center for Biotechnology Information (NCBI) RefSeq assembly (GCF_016772045.1) using Minimap2 (version 2.26-r1175) (5), were removed. Hybrid assembly on Illumina and Nanopore sequencing reads were performed using Unicycler (version 0.4.8) (6), and only one circular contig was generated. Gene annotation, taxonomy check, and average nucleotide identity (ANI) calculation were performed using DFAST (version 1.6.0) and DFAST_QC (version 0.5.5) (7). Phylogenetic analysis of the isolated strain was performed based on the genome-derived full-length 16S rRNA gene using MAFFT online application with the neighbor-joining method (8).

**Fig 1:**
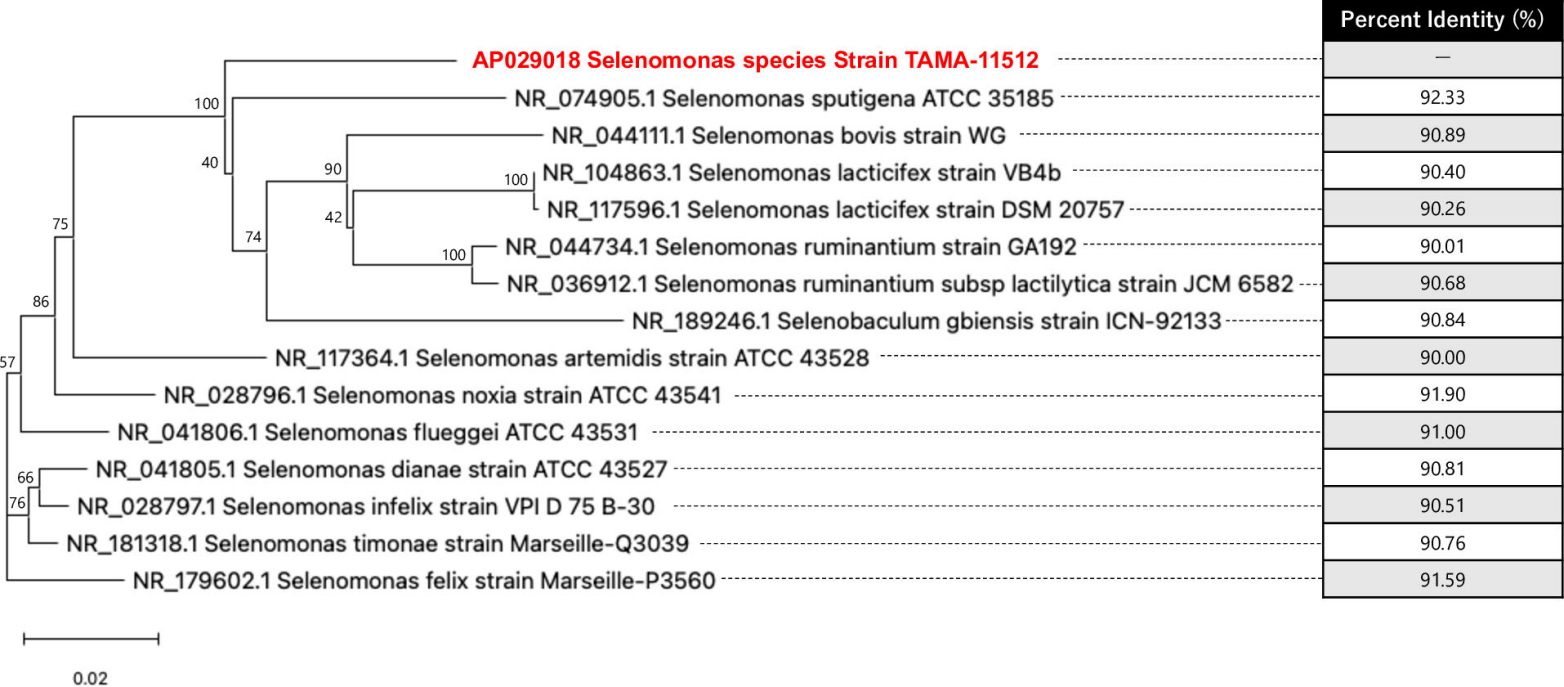
Phylogenetic tree of the *Selenomonas* species strain TAMA-11512 and the other *Selenomonas* genus based on the full-length 16S rRNA gene. Sequences including *Selenomonas* species strain TAMA-11512 (red) were aligned with MAFFT software, and phylogenetic inferences using neighbor-joining method based on the Jukes-Cantor model and bootstrap analysis with 1,000 resampling. Percent identity of nucleotides of 16S rRNA to the query sequence was output by blastn. ATCC, American Type Culture Collection.

The complete genome characteristics are listed in Table 1. The isolated strain was shown to have a similarity to *Selenomonas* genus, but <98.4% identity to the NCBI BLAST 16S rRNA gene sequence database (Fig. 1) and <95% of ANI value (Table 1); the strain TAMA-11512 was considered a novel bacterial species.

**TABLE 1:** Sequencing data and genomic characteristics of *Selenomonas* species strain TAMA-11512

Characteristics	Findings
Illumina sequencing	
Read length (bases)	2 × 150
Number of read pairs	4,739,188
Number of filterd read pairs	4,478,452
Nanopore sequencing	
Number of reads	2,299,715
Read N50 (bases)	5,990
Number of filterd reads	47,714
Average length of filtered reads (bases)	10,479
Complete genome	
Number of contigs	1
Structure	Circular
Total genome length (bp)	2,593,422
GC content (%)	53.9
Average coverage (fold)	
Illumina sequencing	246
Nanopore sequencing	193
Predicted number of coding sequences	2,457
Number of rRNAs	12
Number of tRNAs	52
GenBank accession number	AP029018
*Selenomonas species* (accession number) with high average nucleotide identity values among sequenced species	(%)
*Selenomonas timonae* (GCA_014250475.1)	78.62
*Selenomonas sputigena* (GCA_000208405.1)	78.01
*Selenomonas artemidis* (GCA_000426665.1)	77.18
*Selenomonas montiformis* (GCA_009697385.1)	76.92

## Data Availability

The sequencing data were deposited in the DNA Data Bank of Japan (DDBJ) database, accession number AP029018. Illumina and Nanopore sequencing FastQ data are available in the DDBJ Sequence Read Archive under experiment numbers DRR513996 and DRR456193, respectively.
